# Clinicoradiological diagnosis: Cough-induced transdiaphragmatic intercostal herniation

**DOI:** 10.1259/bjrcr.20190061

**Published:** 2020-09-29

**Authors:** Prema Mohandas, Ahmed O.A. Krim, Jerry Glenn

**Affiliations:** 1 Department of Surgery, Southland Hospital, Kew Road, 9812, Invercargill, Southland, New Zealand; 2 Department of Radiology, Waikato District Health Board, Pembroke Street, Private Bag 3200, Hamilton 3240, New Zealand

## Abstract

Transdiaphragmatic intercostal herniation can occur following blunt or penetrating trauma and is usually associated with rib fractures. It is uncommon and only sporadically reported in literature. We report a case of cough-induced intercostal herniation containing large bowel, on a background of sustaining a blunt chest trauma 25 years prior to presentation. The patient was treated by reducing the hernia followed by surgical repair of the diaphragm and intercostal muscles defect. He was discharged without further complications and remained well at follow-up.

## Introduction

Transdiaphragmatic intercostal herniations (TDIH) is a rare clinical entity characterised by the protrusion of abdominal viscera through the diaphragm and a defect in an intercostal space. They generally develop after trauma, surgery or rarely spontaneously.^1^ TDIH may be recognised during the initial trauma admission. If it is missed during the post-traumatic period, the patient may recover from the initial trauma and remain symptom free, present with chronic thoracoabdominal symptoms or present as an acute emergency with a symptomatic hernia.^2^ During a delayed presentation, the initial trauma responsible for the injury is often forgotten and the diagnosis may not be suspected.^3^ A careful history, physical examination and appropriate imaging are required for the prerequisites of an accurate diagnosis.

This case is unique due to the occurrence of TDIH as a delayed presentation, with a subtle factor precipitating the symptoms.

## Clinical presentation

A 58-year-old male presented to the emergency department with a 10-day history of poorly localised pain on the left side of his chest and upper abdomen, exacerbated by movement. The symptoms preceded a paroxysmal of cough, which precipitated a sudden bulge and ecchymosis over left inferolateral region of the chest. Additionally, he complained of absence of bowel motions for four days and nausea. He had a history of blunt trauma to the chest and abdomen following a high-speed motor bike accident 25 years ago, whereby he suffered rib injuries to his left side. He did not seek medical attention at the time.

**Figure 1.**
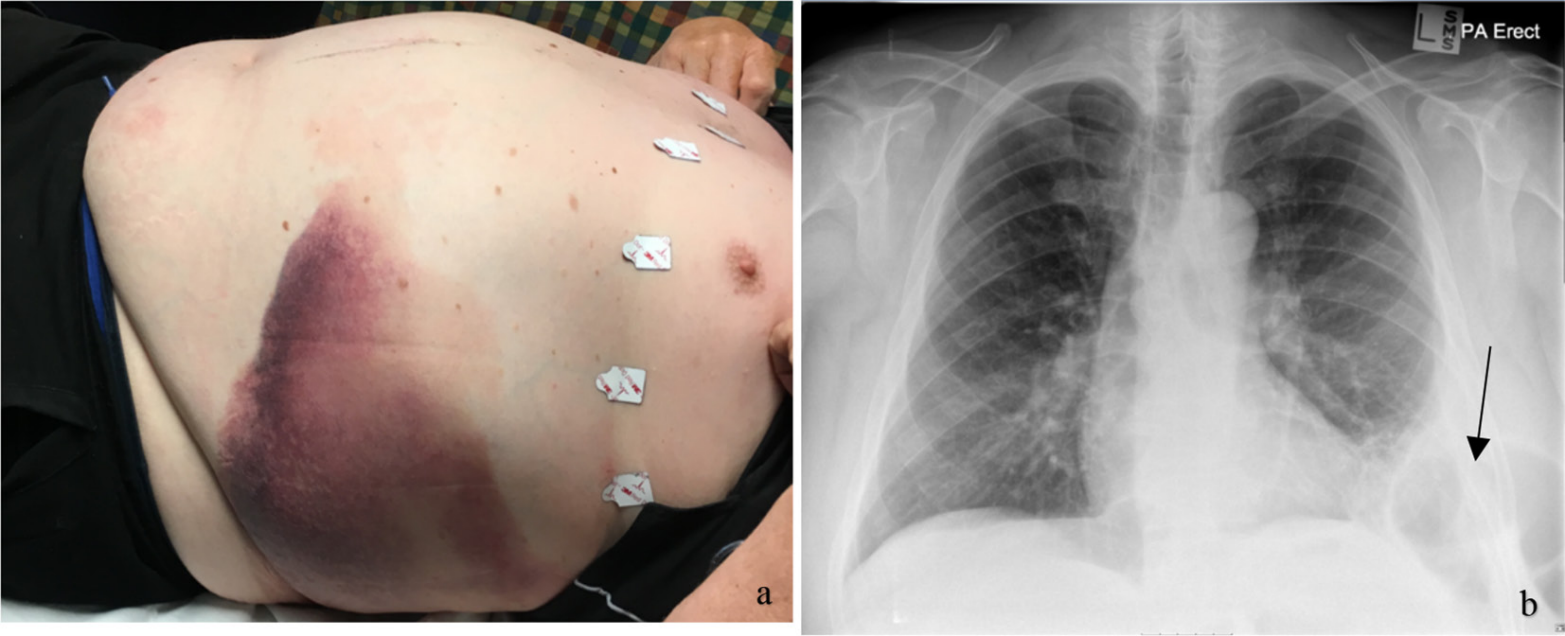
(A) Left upper outer quadrant ecchymosis with bulging of the lower intercostal spaces. (B) A posteroanteriorchest radiography demonstrating a loop of bowel through the intercostal space, suspicious of an intercostal hernia (black arrow).

Physical examination revealed an obese patient who was normotensive, 138/88 mm Hg, tachypnoeic, 20 breaths/min with a pulse rate of 88/min. Examination of the chest revealed ecchymosis (Figure 1a), decreased movement on respiration, absent breath sounds on the left basal zone with dullness on percussion. There was irreducible bulging between the left 9^th^ and 11^th^ intercostal space, which became increasingly evident during a Valsalva manoeuvre. Abdominal examination revealed tenderness in the left upper quadrant with decreased bowel sounds on auscultation. Apart from raised inflammatory marker (CRP 159 mg dl^−1^), all the blood tests were within normal limits. A chest radiograph showed an abnormal lucency in the left lower zone with an appearance of a bowel loop. The lucency was seen extending into the soft tissue of the lateral chest wall concerning for a diaphragmatic and lateral chest wall hernia. The remaining lung fields were clear (Figure 1b).

## Investigations

**Figure 2.**
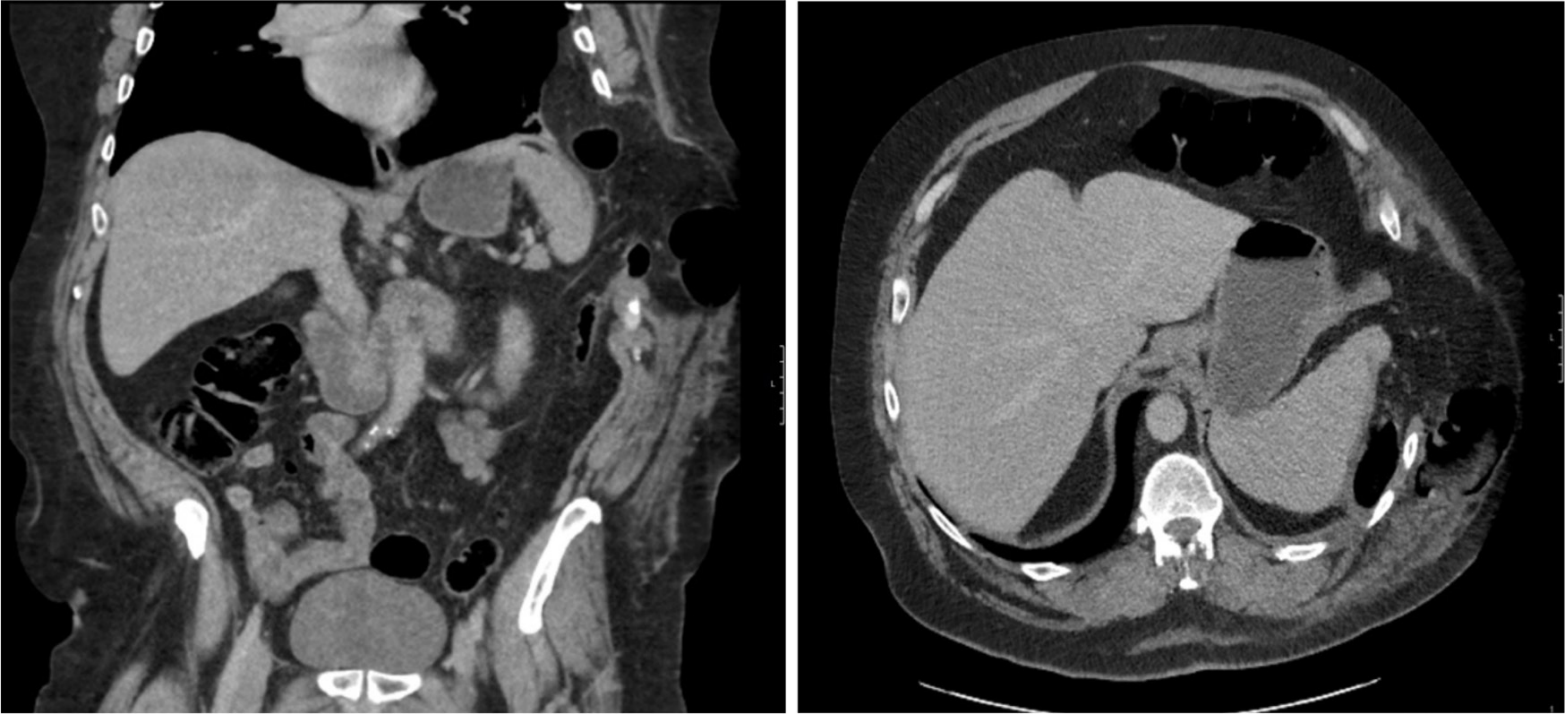
(A) Coronal and (B) axial images revealed a diaphragmatic defect with herniation of bowel into the left intercostal space.

CT of the thorax and abdomen (Figure 2) revealed a large left diaphragmatic hernia containing mesenteric fat and splenic flexure of the colon with small bowel loops. The herniation was through the lateral chest wall between 9th and 10th ribs on the left side with very wide neck measuring approximately 10 cm, in keeping with TDIH. CT scan showed multiple healed posterior rib fractures.

## Treatment

**Figure 3.**
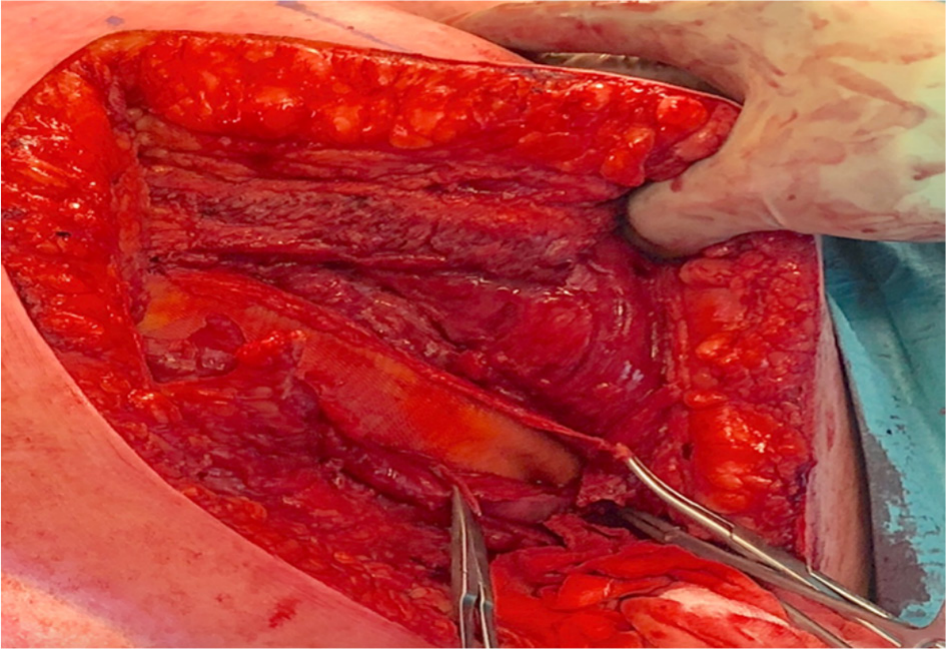
Coated mesh placed under the diaphragm. Clamped artery forceps displaying the edges of the torn diaphragm.

Surgery was expedited and an emergency thoracoabdominal exploration was performed in the operating theatre. The key findings included herniation of the omentum and transverse colon through the diaphragm and the intercostal defect into the subcutaneous tissue. The intra-abdominal contents were reduced although the defect followed by repaired the left diaphragm and the intercostal defects with a coated prolene mesh and sutures (Figure 3).

## Outcome and follow up

**Figure 4.**
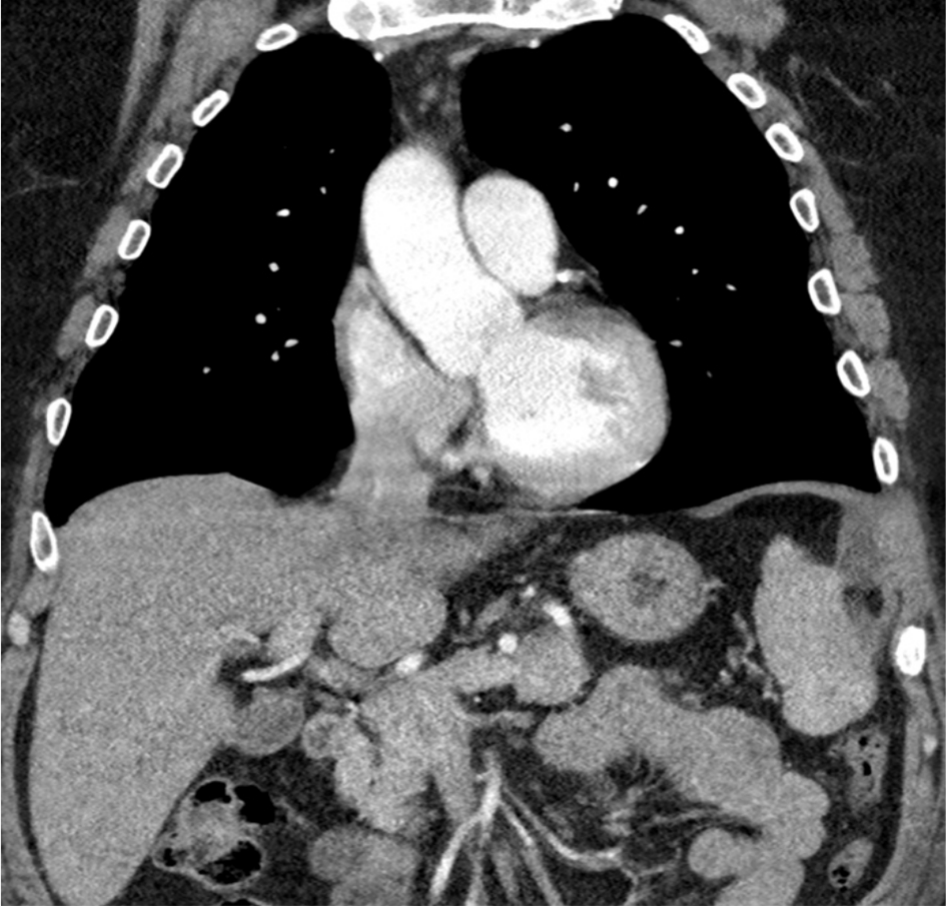
Postoperative CT scan showing reduced hernia with an intact diaphragm.

Postoperative course was uneventful. He had a postoperative CT scan (Figure 4), which showed a small volume seroma at the previous site of the hernia and resolution of the intercostal herniation, he was discharged home without any complications. At the six-month follow-up, the patient remained asymptomatic.

## Differential diagnosis

The leading diagnosis with a previous history of trauma and a sudden onset cough-induced lump is an intercostal hernia, with the contents containing lung or intra-abdominal organs in the event of a concurrent diaphragmatic rupture. Other potential differentials include congenital diaphragmatic hernia, neoplasms, abscess or subcutaneous emphysema, although these differentials in our case are unlikely due to the clinical history and radiological image findings.

## Discussion

The incidence of diaphragmatic rupture is between 0.8 and 1.6% of patient admitted to hospital with blunt trauma.^4^ 75% of rupture is the result from blunt abdominothoracic trauma and the remaining 25% from penetrating injuries.^4^ The following theories have been postulated to explain the mechanism of rupture of blunt injuries. Shearing forces on a stretched membrane, avulsion of the diaphragm from its point of attachment and a sudden increase in the transdiaphragmatic pleuroperitoneal pressure gradient.^5^ This pressure gradient can be increased by coughing, vomiting, exercise or iatrogenic injury.^4^ The commonest site of rupture is the posterolateral surface along the embryonic fusion line as it is the weakest part of the diaphragm.^4^ The left side-has a greater prevalence of rupture 68.5% and the right side 24.2%.^1^ The left-sided predominance can be explained by the hepatic protection of the right side, increased strength of the right hemidiaphragm and the relative weakness of the left due to the lines of embryonic fusion.^3^

In our case, the CT showed herniation of the large bowel through the diaphragmatic and intercostal muscles’ defects. This indicates disruption of the diaphragm at the costal margin as well as weakening or tears in the intercostal muscles following the rib fractures. Detection may be delayed if the rupture is asymptomatic and it may often be overlooked in the presence of life-threatening injuries which take a priority in management. A delayed rupture is possible if the diaphragmatic tissue is devitalised at the time of injury but maintains a barrier until it is weakened.^4^

Investigations for diagnosis should be commenced with a chest radiograph. Although it presents low sensitivity and specificity, its preoperative diagnostic value is further limited in the presence of right-sided diaphragmatic defect or haemothorax.^1^ Pathogenic signs include visualisation of hernial contents above the diaphragm and extension of nasogastric tube in the chest as the stomach is the most frequent herniated organ.^3^ CT abdominal and thoracic scans remain highly specific and sensitive tools for the diagnosis of acute or latent rupture.^6^ The signs include discontinuation of the diaphragm, intrathoracic visceral herniation, lack of visualisation of the diaphragm (absent diaphragm sign), and constriction of the bowel and the herniated organs at the site of the diaphragmatic defect (collar sign).^4^

The morbidity and mortality rate can increase secondary to associated complication of the undiagnosed diaphragmatic injury, mortality ranges from 5–30%.^5^ The complications associated with undiagnosed diaphragmatic rupture may include; bowel herniation, incarceration and strangulation; tension haemothorax secondary to massive bowel herniation; pericardial tamponade from herniation into the pericardial sac.^2^

Classification of injuries involving the costal margin is poorly described in literature and surgical management varies.^7^ Analysis of the costal margin, diaphragm and intercostal muscles can be used to categorise the injuries.^7^ Surgery should address each of the components of injury to maintain a sound repair. In the setting of an acute rupture, resuscitation followed by an operative intervention is mandatory to prevent bowel ischaemia and rupture. A thoracoabdominal incision is preferred in cases of delayed presentation to look for concomitant abdominal injury and for ease of reduction and freeing of adhesions within the chest cavity.^4^ As in our case, repair of the hernia was easily achieved with this incision.

## Conclusion

Delayed presentation of TDIH should be considered in acute sitting of upper abdomen and/or lower chest pain or a sign of bowel obstruction in patients with past history of blunt or penetrating trauma involving either the lower chest or upper abdomen. CT and chest X-ray play an essential role in the diagnosis, and laparotomy with thoracoabdominal incision is advocated as operative choice for acute or delayed presentation.

## Learning points

1. The diagnosis of transdiaphragmatic intercostal herniation (TDIH) is difficult and often missed particularly in delayed presentations following the trauma. This leads to an increased risk of considerable morbidity or mortality from the complication of the hernia.
2. Chest X-ray initially will be helpful although with its limitations. CT scan has become the standard in diagnosis in recognising the signs of direct rupture and indirect signs that are associated with the consequence of the rupture.
3. Accurate diagnosis depends on the combination of clinical and radiological findings.
